# Supplementary material for: Participation of children and adolescents in healthcare, prevention and health research: A dot survey
Source: PLoS One. 2026 Mar 31;21(3):e0345911. doi: 10.1371/journal.pone.0345911 (PMC13037970; doi:10.1371/journal.pone.0345911)
Supplement: Attachment — Submitted filename: Comments for PONE 25 35638.pdf [file pone.0345911.s002.pdf]

Comments:

The study aimed to investigate the age-appropriate participation of children and adolescents in decision-making process in disease prevention, treatment management and decision. The study applied the dot poster method, which is more suitable for engaging children and adolescents, in a sample of 87 paediatric and adult respondents. However, the description of the method would benefit from clearer clarification of overall study design, particularly regarding whether it is qualitative or quantitative. The main study outcomes rely solely on descriptve data (frequencies and counts) merely in a relatively small and biased sample, while statistical inference is lacking. In the disucssion, qualitative data (quotations) were used to illustrate children's opinions, but these data were not described in the methods or presented in the results, raising concerns about consistency. In such a biased population with imbalanced age groups, quantitative results must be interpreted with caution. It should also be cautious that the conclusion may not be statistically generalizable.

An interventional study conducted in a more specific setting (e.g. recurrent infections or chronic conditions such as epilepsy), with defined comparison groups, would provide stronger evidence on the heterogeneity of children's participation in decision-making, its benefits for health outcomes and psychological well-being, and would allow for a more robust conclusion.

General comments:

- It is recommended to begin each paragraph with a clear topic sentence and ensure transitions between paragraphs to enhance the overall coherence and readability.
- The researchers did not clearly articulate the specific research question at the outset, and a corresponding survey question for respondents was also missing. Furthermore, the presentation of analytical results at different levels in the results section makes the manuscript difficult to follow.
- In the introduction, the health benefits of children's participation in health decision-making, the subsequent study design did not adequately reflect or address these aspects.
- If the author aimed to assess the application of dot poster method, a clear rationale for this tool selection should be briefly outlined in the introduction for methodology selection, including why this approach is particularly suitable for peadiatric populations.

Major comments:

- line 141-144: Two age groups are stated here, but it is unclear who constitutes the "two age groups" and how the "four age groups" are ultimated defined. Earlier (line 64-70), paediatric population was divided into three age groups. Could the

authors please explain why the three-group classification was not used in the statistical analysis consistently?

- line 145: Could the authors state the exact question that was psoed to respondents? This would help guide the reader and improve clarity and reproducibility.
- line 198: The table requires significant improvements in format, content, and clarity to enhance readability and interpretability. First, the table has overly complex structure, which crosses respondents' age groups with the age of decision-making target. This makes the interpretation difficult (e.g. a 7-year-old respondent giving an opinion about 12-year-olds' decision making). Second, it is unclear at times whether the statement refers to the respondent themselves or the children/adolescents being considered as decision-makers. Third, the row labels are repetitive and lengthy. Using abbreviations or short labels is recommended to improve readability. Similar issues are noted in Table 2 and 3.
  In terms of the data interpretation, some cells have very few respondent (e.g. n=4) but still report percentages as 75%. This can be misleading and exaggerate the weight of those responses.
- The dot poster method can be used in group settings. Could the authors clarify whether the responses collected independently, and whether any measures were taken to minimize the influence of one participant's responses on others?
- line 159-164: The method section should provide a detailed description of the statistical analysis method is required, including both descriptive and any planed analytical approaches.
- line 169-: In the result section, the researchers report a small sample size of respondents. Could the authors clarify how the sample size was determined and discuss whether it is adequete to support the analyses and the representativeness of a larger population?

Minor comments:

- line.24-25: The clarity of the sentence could be improved. A revised version is: Traditionally, parents and doctors make decisions for children, as children are often considered lacking in cognitive maturity and the ability to make decisions
- line 54 – 56: The sentense contains some duplications, which makes it feel slightly redundant. Rephrasing the sentence to reduce repetition would improve clarity and flow.
- line 169: Since style conventions generally recommend not starting a sentence with numerals, revision is recommended.
- line 176: Why an "in-between answers" option was included only in on-site survey?
