# Supplementary material for: Participation of children and adolescents in healthcare, prevention and health research: A dot survey
Source: PLoS One. 2026 Mar 31;21(3):e0345911. doi: 10.1371/journal.pone.0345911 (PMC13037970; doi:10.1371/journal.pone.0345911)
Supplement: Attachment — Submitted filename: Response to Reviewers.docx [file pone.0345911.s004.docx]

Dear editor, dear reviewers,

Thank you very much for the comprehensive comments on our manuscript. We have carefully reviewed all comments and implemented them as far as possible and we think that the manuscript has been significantly improved by the reviews. We hope that the manuscript now meets the requirements for publication in PLOS ONE.

Best regards, for the authors, Yvonne Jordan and Neeltje van den Berg

Journal Requirements:

When submitting your revision, we need you to address these additional requirements.

1.Please ensure that your manuscript meets PLOS ONE's style requirements, including those for file naming. The PLOS ONE style templates can be found at

<https://journals.plos.org/plosone/s/file?id=wjVg/PLOSOne_formatting_sample_main_body.pdf> and

[https://journals.plos.org/plosone/s/file?id=ba62/PLOSOne_formatting_sample_title_authors_affiliations.pdf](https://track.editorialmanager.com/CL0/https:%2F%2Fjournals.plos.org%2Fplosone%2Fs%2Ffile%3Fid=ba62%2FPLOSOne_formatting_sample_title_authors_affiliations.pdf/1/010f0198ec09c5fb-de07d400-981b-4f7c-a55c-1f850f4a81ea-000000/Ii6QYsZ9goXi5njrtBHHr399OOr5iv4qx1azW8R_5Aw=225)

We have carefully reviewed the document and made corrections.

2. In the ethics statement in the Methods, you have specified that verbal consent was obtained. Please provide additional details regarding how this consent was documented and witnessed, and state whether this was approved by the IRB

See lines 179-180: There was no written documentation of consent.

Ethical approvement: see reviewer 1, comment 2

3. We note that you have indicated that there are restrictions to data sharing for this study. PLOS only allows data to be available upon request if there are legal or ethical restrictions on sharing data publicly. For more information on unacceptable data access restrictions, please see <http://journals.plos.org/plosone/s/data-availability#loc-unacceptable-data-access-restrictions>.

Before we proceed with your manuscript, please address the following prompts:

a) If there are ethical or legal restrictions on sharing a de-identified data set, please explain them in detail (e.g., data contain potentially identifying or sensitive patient information, data are owned by a third-party organization, etc.) and who has imposed them (e.g., a Research Ethics Committee or Institutional Review Board, etc.). Please also provide contact information for a data access committee, ethics committee, or other institutional body to which data requests may be sent.

b) If there are no restrictions, please upload the minimal anonymized data set necessary to replicate your study findings to a stable, public repository and provide us with the relevant URLs, DOIs, or accession numbers. For a list of recommended repositories, please see

<https://journals.plos.org/plosone/s/recommended-repositories>. You also have the option of uploading the data as Supporting Information files, but we would recommend depositing data directly to a data repository if possible.

We will update your Data Availability statement on your behalf to reflect the information you provide.

We have reviewed the data and conditions again and have come to the conclusion that we can make the data available. WE will provide the data as supporting information file.

4. Please include your full ethics statement in the ‘Methods’ section of your manuscript file. In your statement, please include the full name of the IRB or ethics committee who approved or waived your study, as well as whether or not you obtained informed written or verbal consent. If consent was waived for your study, please include this information in your statement as well.

See lines 180-184:

In anonymous surveys, there is, according to the guidelines of the German Research Foundation (DFG) and the professional code of conduct for physicians in the Federal State of Mecklenburg-Western Pomerania, no need to consult the local Ethics Committee.

This was confirmed by the Ethics Committee of the University Medicine Greifswald.

Reviewers' comments:

Reviewer #1: Thank you for the opportunity to review this important manuscript. This study is a good example of how simple, easy-to-implement methods can still impart meaningful findings. My suggestions to improve the manuscript are the following:

Background

1. You raise three distinct areas where children and adolescents can be involved in decision-making. To make these distinctions clearer, it would be helpful to have subheadings in the background section.

We included subheadings in the background section.

Methods

2. Was research ethics board approval obtained for this study? If so, state this and provide the REB number from your institution. If not, state this, and explain the greater context of the study as quality improvement to justify the lack of REB approval.

Since the data, assessed in this study, were anonymous, the project was, according to the guidelines of the German Research Foundation (DFG) and the professional code of conduct for physicians in the Federal State of Mecklenburg-Western Pomerania, not subject to the consultation requirement of the local Ethics Committee. We submitted the statement of our Ethics Committee to this fact to PLOS One.

We added the following text to the methods section (lines 180-184):

In anonymous surveys, there is, according to the guidelines of the German Research Foundation (DFG) and the professional code of conduct for physicians in the Federal State of Mecklenburg-Western Pomerania, no need to consult the local Ethics Committee. This was confirmed by the Ethics Committee of the University Medicine Greifswald.

3. Line 143-144: The four age groups are listed in your tables, but they should also be shared in the text.

We adapted the text in the methods section (lines 154-157):

Questions 1 to 4 should be answered for 2 age groups: children under 13 years and adolescents aged 13 to 18 years (Fig 1). The respondents were asked for their age and assigned to four age groups: ≤6 years (pre-school children), 7-12 years (school children), 13-17 years (adolescents), >17 years (adults).

4. Line 153: Since North America and Europe use different date formats, it would be better to share the date as October 3, 2024 to improve clarity.

We adopted your suggestion (line 168)

5. I am unsure of how data was actually collected for the survey. Who completed the survey on the poster, and who used the QR code to access the online version and how was this decided?

Respondents could choose between the survey modes. We adapted the text in the methods section to clarify this (lines 165-166):

For participants who preferred this survey mode, an online survey with the same questions and answer categories was created in addition to the poster.

6. When did the research team obtain qualitative feedback from participants and how was it documented (e.g., field notes, verbatim transcription, audio recording, etc.)?

We added the following text to the methods section (lines 192-194):

During the data collection, we documented spontaneous statements from participants with the aim of supporting the interpretation of the survey (field notes). Therefore, the statements are included in the discussion.

Results

7. Table 1: Percentages should have a period instead of a comma for English-language publications

We corrected this.

Discussion

8. Rather than presenting your qualitative data in the discussion, it should be presented in the results section and analyzed appropriately, even if just using a simple content analysis. The methods for doing so should be shared in the methods section. It is clear from these quotations that there is meaningful data here which deserves to be analyzed and could help contextualize the quantitative data.

Thank you for this comment. We agree, that the statements of the respondents add meaningful information to the results of the analysis. However, the statements were collected for use in interpreting the results; unfortunately, the documentation is not suitable for qualitative analysis (see also comment 6).

To make better use of this type of information in the future, we will adapt the qualitative survey methods in future surveys to enable us to conduct a real qualitative analysis and we ask for your understanding that we can only use the statements for discussion this time.

Overall

9. Approaches to engagement of children and adolescents in health vary greatly based on geographical location. In my experience, German clinicians and researchers have tended to be more conservative toward this type of engagement than in other countries such as Canada. Rather than discussing child and adolescent engagement globally, your manuscript would be stronger by contextualizing child and adolescent engagement broadly, and then reflecting on your results in a German or EU context in the discussion section.

Thank you very much for this comment, which we fully agree with. We added the following text in the conclusion of the discussion section (lines 392-394):

Research results and practical experience are necessary to bring about a cultural change towards greater participation in Germany, where the approach to participation of children and young people has so far been rather conservative.

Reviewer #2: The study aimed to investigate the age-appropriate participation of children and adolescents in decision-making process in disease prevention, treatment management and decision. The study applied the dot poster method, which is more suitable for engaging children and adolescents, in a sample of 87 paediatric and adult respondents. However, the description of the method would benefit from clearer clarification of overall study design, particularly regarding whether it is qualitative or quantitative. The main study outcomes rely solely on descriptve data (frequencies and counts) merely in a relatively small and biased sample, while statistical inference is lacking. In the disucssion, qualitative data (quotations) were used to illustrate children’s opinions, but these data were not described in the methods or presented in the results, raising concerns about consistency. In such a biased population with imbalanced age groups, quantitative results must be interpreted with caution. It should also be cautious that the conclusion may not be statistically generalizable.
An interventional study conducted in a more specific setting (e.g. recurrent infections or chronic conditions such as epilepsy), with defined comparison groups, would provide stronger evidence on the heterogeneity of children’s participation in decision-making, its benefits for health outcomes and psychological well-being, and would allow for a more robust conclusion.

10. However, the description of the method would benefit from clearer clarification of overall study design, particularly regarding whether it is qualitative or quantitative.

We added the following sentence in the method section (line 131):

This is an observational, cross-sectional study on the basis of quantitative data.

11. The main study outcomes rely solely on descriptve data (frequencies and counts) merely in a relatively small and biased sample, while statistical inference is lacking.

We agree to your comment, the number of observations was too small to conduct multivariate analyses.

12. In the disucssion, qualitative data (quotations) were used to illustrate children’s opinions, but these data were not described in the methods or presented in the results, raising concerns about consistency.

We added the following sentence to the method section (lines 192-194):

During the data collection, we documented spontaneous statements from participants with the aim of supporting the interpretation of the survey (field notes). Therefore, the statements are included in the discussion.

13. In such a biased population with imbalanced age groups, quantitative results must be interpreted with caution. It should also be cautious that the conclusion may not be statistically generalizable.

We addressed this concern in the discussion section under “Limitation” (lines 353-354).

In the Conclusion, we added something to the last sentence (lines 394-397):

Despite some methodological limitations, the results provide initial insights into the population's views on the participation of children and adolescents in the healthcare system and form a basis **for further research to improve** the opportunities for children and adolescents to participate in the healthcare system.

14. An interventional study conducted in a more specific setting (e.g. recurrent infections or chronic conditions such as epilepsy), with defined comparison groups, would provide stronger evidence on the heterogeneity of children’s participation in decision-making, its benefits for health outcomes and psychological well-being, and would allow for a more robust conclusion.

Thank you for this comment. We think, the results of our study can be part of a basis for further research, including interventional studies (see our reply 13).

**General comments:**

15. It is recommended to begin each paragraph with a clear topic sentence and ensure transitions between paragraphs to enhance the overall coherence and readability.

We carefully read and adapted the manuscript in view of this comment.

16. The researchers did not clearly articulate the specific research question at the outset, and a corresponding survey question for respondents was also missing.

We added the concrete research question at the end of the background section (lines 128-129):

The research question of this study is: To what extent should children and adolescents be involved in decisions regarding health care, prevention, and health research?

The question asked to the participants can be found in lines 141-143:

Respondents were asked to answer the question “In which areas of healthcare should children and adolescents have a say or be involved? Are there differences between younger children and adolescents?” by placing coloured, round self-adhesive dots on the poster next to the categories that most closely reflected their opinion

17. Furthermore, the presentation of analytical results at different levels in the results section makes the manuscript difficult to follow.

The results section is divided in 4 parts: sample size, healthcare (treatment and therapy), prevention, and health research. Each part of the results section has its own results table. We hope that the results section is so clearly structured.

18. In the introduction, the health benefits of children’s participation in health decision-making, the subsequent study design did not adequately reflect or address these aspects.

Thank you for this valuable comment. We added the following sentence to create a better transition between the background and the research question (lines 119-121):

Overall, there is a research gap regarding the opinions of children, adolescents and their parents on participation in healthcare, prevention and health research, especially with regard to different age groups.

19. If the author aimed to assess the application of dot poster method, a clear rationale for this tool selection should be briefly outlined in the introduction for methodology selection, including why this approach is particularly suitable for peadiatric populations.

We added the following text in the methods section (lines 134-138):

The dot poster method was initially developed for surveys to explore the shopping behavior of visitors of farmers’ markets [20,21]. A dot poster survey to gain insight into young patients’ attitudes about mental health showed positive results with respect to the feasibility of this method in young persons [22].

**Major comments:**

20. line 141-144: Two age groups are stated here, but it is unclear who constitutes the “two age groups” and how the “four age groups” are ultimated defined. Earlier (line 64-70), paediatric population was divided into three age groups. Could the authors please explain why the three-group classification was not used in the statistical analysis consistently?

Thank you for this comment! We have two kinds of age-group-stratifications:

1. The participants of the study (respondents). We divide them into 4 age groups on the basis of developmental stages: ≤6 years (pre-school children), 7-12 years (school children), 13-17 years (adolescents), >17 years (adults).
2. The participants had to answer the questions for 2 age groups: ≤ 12 years and 13 to 17 years

Because this scheme has a large complexity, we adapted the text in the methods section to clarify this (lines 154-157):

Questions 1 to 4 should be answered for 2 age groups: children under 13 years and adolescents aged 13 to 18 years (Fig 1). The respondents were asked for their age and assigned to four age groups: ≤6 years (pre-school children), 7-12 years (school children), 13-17 years (adolescents), >17 years (adults).

21. line 145: Could the authors state the exact question that was psoed to respondents? This would help guide the reader and improve clarity and reproducibility.

We have added the question (lines 141-143). The entire question is:

In which areas of the healthcare system (health care, prevention, and health research) should children and adolescents have a say or be involved? Are there differences between younger children and adolescents?

22. line 198: The table requires significant improvements in format, content, and clarity to enhance readability and interpretability. First, the table has overly complex structure, which crosses respondents’ age groups with the age of decision-making target. This makes the interpretation difficult (e.g. a 7-year-old respondent giving an opinion about 12-year-olds’ decision making). Second, it is unclear at times whether the statement refers to the respondent themselves or the children/adolescents being considered as decision-makers. Third, the row labels are repetitive and lengthy. Using abbreviations or short labels is recommended to improve readability. Similar issues are noted in Table 2 and 3.

We adapted the tables.

23. In terms of the data interpretation, some cells have very few respondent (e.g. n=4) but still report percentages as 75%. This can be misleading and exaggerate the weight of those responses.

We added the following text to the limitations section of the discussion: (lines 361-364)

The number of respondents was rather small. Due to the division of the respondents in different age groups and the fact that they had to answer the statements for 2 age groups, some cells have a very small number or even zero entries. This must be taken into account when interpreting the results.

24. The dot poster method can be used in group settings. Could the authors clarify whether the responses collected independently, and whether any measures were taken to minimize the influence of one participant’s responses on others?

We added the following text to the limitations section of the discussion: (lines 369-370):

To minimize the influence of one participant’s responses on others, all participants were informed and guided separately.

25. line 159-164: The method section should provide a detailed description of the statistical analysis method is required, including both descriptive and any planed analytical approaches.

We adapted the description of the statistical analysis (lines 184-186):

The dots in in the different cells of the poster as well as the number of respondents in each age group were counted, frequencies and proportions were calculated.

26. line 169-: In the result section, the researchers report a small sample size of respondents. Could the authors clarify how the sample size was determined and discuss whether it is adequete to support the analyses and the representativeness of a larger population?

Thank you for this important comment. We added the following sentence to the methods section (lines 169-171):

The dot-poster was presented at the stand of the University Medical Center Greifswald. It was not possible to calculate a sample size in advance because it was not known how many visitors would visit this stand. Possible respondents were visitors who came close enough to the poster to be addressed.

The limitation part of the Discussion includes the following text to this topic (lines 353-358):

This type of sampling carries the risk of selection bias or self-selection bias, therefore, the sample is probably not representative for the general population. In our study, the majority of respondents were over 18 years of age, and it can be assumed that they were mainly people interested in science and possibly people with a higher level of education/socioeconomic status who took part, as well as healthy rather than seriously ill children, although we cannot verify this from the poster.

We added the following sentence to address the adequateness of the sample (lines 358-360):

However, the sample can provide initial insights into the opinions of different age groups in the population regarding the participation of children and young people in the health sector.

**Minor comments:**

27. line.24-25: The clarity of the sentence could be improved. A revised version is: Traditionally, parents and doctors make decisions for children, as children are often considered lacking in cognitive maturity and the ability to make decisions

thank you very much for your suggestion, which we have adopted.

28. line 54 – 56: The sentense contains some duplications, which makes it feel slightly redundant. Rephrasing the sentence to reduce repetition would improve clarity and flow.

We adapted the sentence (lines 55-56):

However, while adult patients are encouraged to actively participate in decisions about their medical care, this is not yet the case for children and adolescents

29. line 169: Since style conventions generally recommend not starting a sentence with numerals, revision is recommended.

We adapted the sentence (line 195):

A total of 87 people took part in the survey.

line 176: Why an “in-between answers” option was included only in on-site survey?

We adapted the sentence to clarify this (lines 203-204):

The option for “intermediate answers” was not available in the online survey because one answer had to be selected and there was no opportunity for changes or free text.
