# Supplementary material for: Participation of children and adolescents in healthcare, prevention and health research: A dot survey
Source: PLoS One. 2026 Mar 31;21(3):e0345911. doi: 10.1371/journal.pone.0345911 (PMC13037970; doi:10.1371/journal.pone.0345911)
Supplement: Attachment — Submitted filename: Response_to_reviewers_auresp_2.docx [file pone.0345911.s005.docx]

Dear Editor, dear Reviewer,

Thank you very much for your constructive and valuable comments. We have implemented them as best we could and believe that the paper has gained more quality as a result. We hope that the paper now meets all the requirements for publication in PLOS ONE.

Best regards, for the authors, Yvonne Jordan and Neeltje van den Berg

Point-to-point reply:

Reviewer #3: 

Children aged six and younger were included. Most children in this age group are not literate and very likely unable to read and understand the words on the dot poster. What methods were implemented to ensure these children provided valid responses? How likely is it that these responses are driven by the parents?

“Prevention” is one of the decision areas on the dot survey, but there is no context provided. Was this defined during the briefing? How was it described? Were descriptions of the levels of youth involvement provided (especially for younger children)? These details should be included in Methods.

We expanded the text in the method section (lines 158-162):

The respondents received an age-appropriate briefing both to explain the three topics and the dot poster method. Especially with young children, examples were used to explain what healthcare, prevention, and health research are. A child-friendly example of prevention is that brushing your teeth prevents toothaches., This was not standardized but adapted to the child's level of knowledge. After the briefing, the respondents were given 6 sticky dots of the same colour, with the colour of the sticky dots depending on the age of the respondents.

We included the following sentence in the discussion to explain how we dealt with small children (lines 375-377):

Especially with small children, the topics on the poster were explained in child-friendly language, and the child was always spoken to directly, not the parents, in order to obtain the child's own answer as much as possible.

Figure 1 suggest that participants were asked, “In which areas of healthcare should children and adolescents participate or be involved?”, but in Methods (lines 141-143), the wording of the question is different: “In which areas of the healthcare system (healthcare, prevention, and health research) should children and adolescents have a say or be involved? Are there differences between younger children and adolescents?” These should match exactly what the participants would have read on the dot poster.
We adapted the text in the methods section (lines 141-143):

Respondents were asked to answer the question “In which areas of the healthcare system should children and adolescents participate or be involved?”

Minor issues to address:
Several sentences begin with a number. These should be rewritten.
We changed the respective sentences:

Line 35: “87 people…”,

A total of 87 people took part in the survey.

“30 of the respondents”

Thereof, 30 were under 18 years old (34.5%).

Line 195: “5 of the respondents”

(Now line 199) Thereof, 5 (5.7%) answered the questions online

Line 197: “19 respondents”

(Now line 202) Of the 87 respondents, four (4.6%) were six years old or younger (see table 1), 19 respondents (21.8%) were between seven and twelve years old and seven respondents (8.0%) were between 13 and 17 years old (Table 1).

Line 242: “57.9% of respondents”
(Now line 240) Among respondents aged 13 to 17, 57.1% considered that children should have the opportunity ..

Reference/naming the data collection method should be consistent throughout the manuscript. Multiple variations are included. It is currently referred to as “dot poster method” (lines 32, 132, 133), “dot poster survey” (lines 136, ), “poster-dot survey” (line 138, line 283), “dot-poster” (lines 146, 169), “dot poster” (line 191 – title of Figure 1; heading for tables 2, 3, and 4)), “poster dot survey” (notes for Tables 2, 3, and 4), and “poster dot method” (lines 366, 371, 379).

We rephrased the text with respect tot he data collection method:

We wrote „Dot poster method“ where the data collection method is meant, and „dot poster“ when the poster itself is meant.

Line 185: The word “in” is repeated (“The dots in in the different cells…”).

We corrected this.

Reviewer #4: Thank you for addressing the Comments of the reviewers. I have only two comments.
1. The sample size is too small. Can you repeat the study with a bigger sample size?

2. And add to that study an intervention component: See if involving the children in decision making improves a)cooperation and b)outcomes.

We adapted the conclusion (lines 401-405); the adjustments are underlined): Despite some methodological limitations, the dot poster method was feasible and the results provide initial insights into the population's views on the participation of children and adolescents in the healthcare system. This is a basis for further research with larger sample sizes and interventional components to improve the opportunities for children and adolescents to participate in the healthcare system.
